# Incisional paresthesia following clavicle plate fixation: does it matter to patients?

**DOI:** 10.1186/s12891-021-04770-z

**Published:** 2021-11-03

**Authors:** Valérie Lemieux, Soheil Afsharpour, Diane Nam, Amr Elmaraghy

**Affiliations:** 1 Department of Surgery – Division of Orthopaedic Surgery, University of Toronto, 149 College Street, Toronto, Ontario M5T 1P5 Canada; 2 Sunnybrook Health Sciences Centre, Toronto, Canada; 3 Royal College of Surgeons in Ireland University of Medicine and Health Sciences, Dublin, Ireland; 4 Division of Orthopaedic Surgery, St. Joseph’s Health Centre, Unity Health Toronto, Toronto, Canada

**Keywords:** Paresthesia, Numbness, Iatrogenic supraclavicular nerve injury

## Abstract

**Background:**

Operative management of clavicle fractures is increasingly common. In the context of explaining the risks and benefits of surgery, understanding the impact of incisional numbness as it relates to the patient experience is key to shared decision making. This study aims to determine the prevalence, extent, and recovery of sensory changes associated with supraclavicular nerve injury after open reduction and plate internal fixation of middle or lateral clavicle shaft fractures.

**Methods:**

Eighty-six patients were identified retrospectively and completed a patient experience survey assessing sensory symptoms, perceived post-operative function, and satisfaction. Correlations between demographic factors and outcomes, as well as subgroup analyses were completed to identify factors impacting patient satisfaction.

**Results:**

Ninety percent of patients experienced sensory changes post-operatively. Numbness was the most common symptom (64%) and complete resolution occurred in 32% of patients over an average of 19 months. Patients who experienced burning were less satisfied overall with the outcome of their surgery whereas those who were informed of the risk of sensory changes pre-operatively were more satisfied overall.

**Conclusions:**

Post-operative sensory disturbance is common. While most patients improve, some symptoms persist in the majority of patients without significant negative effects on satisfaction. Patients should always be advised of the risk of persistent sensory alterations around the surgical site to increase the likelihood of their satisfaction post-operatively.

**Supplementary Information:**

The online version contains supplementary material available at 10.1186/s12891-021-04770-z.

## Background

Fractures of the clavicle are common, accounting for 2.6–4% of all fractures and 44% of fractures involving the shoulder girdle [1, 2]. They most often occur in young males and in the middle third of the bone [1, 3–6]. Traditionally, these fractures were treated non-operatively, but studies have since demonstrated improvement in union rates, less fracture deformity, and earlier return to function, following surgical stabilization [7–11]. Consequently, a trend towards operative management has emerged with good overall outcomes [12, 13]. Controversy persists as cost-effectiveness of surgical intervention remains unclear and recent meta-analyses failed to demonstrate clinically significant benefits in patient function [14, 15]. The lack of clear operative benefits and well-defined indications necessitates improved understanding of the patient experience in order to deliver optimal patient-centered care [16].

Post-operative sensory disturbances have been variably linked to patient experience in other orthopaedic procedures [17, 18]. The supraclavicular nerve is at risk of injury during clavicle fixation. It pierces through the platysma crossing the clavicle 97% of the time, most commonly over the lateral two thirds, to provide sensation to the anteromedial shoulder and upper chest wall [19–21]. It may have two or three branches and their courses have notable variability [20], with one case report of a transosseous path [22], making its identification difficult. Anatomic studies have reported common safe zones within 2.7 cm of the sternoclavicular joint and 1.9 cm of the acromioclavicular joints thus leaving the nerve vulnerable to injury during the approach to the most common fracture pattern, i.e., involving the middle third diaphysis [20].

Reports of post-operative sensory disturbance following longitudinal approach along the clavicle are heterogenous in definition and measured time points. Incidence varies widely from 6 to 90% (Appendix I). There is a consistent trend of improvement over time, though without full resolution for all patients [11, 19, 23–36]. Previous groups have demonstrated good outcomes despite high prevalence of numbness leading them to refute correlation between numbness and patient satisfaction [19, 23, 31]; whereas You et al. [29] concluded that conventional approaches led to discomfort in 74.3% of their patients. The impact of sensory disturbances on patients’ outcomes has not been well elucidated. The aim of this study was to report the patients’ subjective experience of post-operative incisional paresthesia.

## Methods

### Study design

This study presents the results of a patient-satisfaction survey of adult patients (Age ≥ 18 years) who underwent an early (< 6 weeks) primary open reduction and internal plate fixation of middle or lateral clavicle shaft fractures between 2009 and 2019. Patients were identified retrospectively using surgical billing codes (*n* = 397). A total of 169 patients were found to be eligible following individual chart review based (Appendix II). Institutional Review Board approved this study prior to patient enrollment. All surgical procedures were completed a minimum of 14 months prior to the survey completion. Patients who had undergone previous/secondary surgical intervention on the ipsilateral clavicle/anteromedial chest/shoulder were excluded to minimize impact of recurrent supraclavicular nerve injury (thus excluding patients who required revision surgery for symptomatic non-union, hardware removal, or other).

### Surgical approach and post-operative protocols

Surgical procedures took place at two sites: an academic, level-1 trauma center (involving multiple surgeons) and a community hospital (single-surgeon). All surgical procedures were performed through an oblique incision along the length of the clavicle overlying the fracture site; the supraclavicular nerve branches were identified and protected when possible. A subperiosteal elevation was completed to expose the fracture and in all cases, an anatomic reduction was achieved to restore the length alignment and rotation. The fascia was closed to obtain full enclosure of the plate, followed by standard closure of the subcutaneous and skin layers with absorbable sutures. The post-operative patient care including immobilization period, analgesia provided, and rehabilitation protocols were individualized as per surgeon preference. The study agglomerated patients from multiple surgeons to reflect “real world” practice variability.

Patients were contacted by mail or telephone directly by the authors, and written or verbal informed consent was obtained prior to completion of the survey. Questions assessed operative consent, the nature and extent of symptoms, functional outcomes, and satisfaction with overall using a numerical rating scale (Appendix III). Eighty-six responses were obtained (67% response rate) and data was anonymized prior to analysis.

### Statistical analysis

Chi-square test was applied to categorical data and paired two-tailed Student’s t-test applied to parametric scale variables. Significance was defined as a *p*-value < 0.05. Cohort-wide associations between questions were found by using the Pearson correlation coefficient. Authors completed the data analysis.

## Results

Paresthesia is a common post-operative symptom; 90% of patients attested they experienced some degree of sensory disruption post-operatively though 32% of these patients saw complete resolution of their symptoms at an average of 19 months (Table 1). There was no correlation between patient age or gender and incidence of post-operative sensory changes. Patients without symptoms and those who experienced full resolution tended to be slightly more satisfied overall than those with persistent symptoms though this finding did not reach significance (9.2/10 vs. 8.6/10, *p*-value 0.08). Earlier resolution of symptoms did not lead to greater satisfaction. The shortest follow-up interval was 14 months. Those who completed the survey > 2 years after surgery did not have a significantly higher rate of symptom resolution or difference in symptom severity.

**Table 1:** Patient Characteristics, Symptoms, and Satisfaction Summary

**Patient Characteristics**				
Total enrollment		86		
Gender	Men	87% (*n* = 75)		
	Women	13% (*n* = 11)		
Average age		41.3 years		
	Men	41.4 years (range: 18–75)		
	Women	40.5 years (range 26–55)		
Months elapsed since procedure		49.4 months (4.1 years) (range 14–122)		
**Symptom Characteristics**				
Incidence of post-operative sensory changes		90%		
Nature of symptoms		(*n* = 77*)		
	Numbness	64%		
	Burning	12%		
	Pain	4%		
	Tingling	3%		
Rate of complete resolution		32% (*n* = 25)		
Median time to resolution		12 months (range 3–84 months)		
Severity of ongoing symptoms		4/10		
Average size of affected area		36.6 cm^2^ (+/− 26.9 cm^2^)		
*Ongoing Symptoms*				
Size resolution:				
	Improving	69%		
	Stable	31%		
	Worsening	0%		
Intensity resolution:				
	Improving	69%		
	Stable	27%		
	Worsening	4%		
Patient Satisfaction**				Correlation significance
Overall		8.8		
Range of motion		8.8		p-value = < 0.05***
	Women		9.2	*p-value = 0.06*
	Men		8.7	
	Age < 25 yrs.		9.6	*p-value = 0.4*
	Age > 55 yrs		8.7	
Shoulder strength		8.2		p-value = < 0.05***
	Women		8.2	*p-value = 0.5*
	Men		8.6	
	Age < 25 yrs.		9.4	*p-value = 0.06*
	Age > 55 yrs		7.6	
Scar appearance		8.5		p-value = < 0.05***
	Women		8.3	*p-value = 0.7*
	Men		8.5	
	Age < 25 yrs		8.9	*p-value = 0.2*
	Age > 55 yrs		9.5	

*Patients were given the option of reporting several symptoms

** Patients were asked to rate their satisfaction on a numerical rating scale of 0–10 where 10 is most satisfied

***Designates a statistically significant finding

Numbness was the most common form of paresthesia, affecting 64% of those who reported symptoms, and burning was the second most common (12%). The nature or number of symptoms did not correlate with likelihood of resolution or improvement in size or intensity. Burning was significantly correlated with lower patient satisfaction compared to patients who experienced other symptoms (7/10 vs. 8.6/10, *p*-value 0.006). On average, reported symptom severity was moderate (4/10) though there was high variability. However, there was no correlation between severity of symptoms and overall patient satisfaction.

In keeping with known inconsistent nerve course, the size of the affected area varied widely with an average of 37 cm^2^ (range 5-115 cm^2^). The size of the affected area did not correlate with likelihood of symptom improvement, patients’ overall satisfaction, or their subjective shoulder strength.

All patients who reported a reduction in the size of the affected area also noted an improvement in intensity. The three patients who reported a worsening in intensity all had multiple symptoms and significantly lower overall satisfaction scores (*p*-value 0.03). Overall, patients who experienced multiple symptom types did not report worse satisfaction outcomes.

Over half (57%) of our surveyed patients recalled being informed of the risk of sensory changes pre-operatively. Those who were warned were significantly more satisfied with the overall outcome of their surgery (9.2/10 vs. 8.3/10, *p*-value 0.02) without correlation to degree of resolution of their symptoms.

Overall, patients were generally pleased with the global outcome of their procedure, their range of motion, ipsilateral shoulder strength, and scar appearance (Table 1); only 8% of patients ranked their overall outcome below 5/10, where 10 is completely satisfied. However, 14% of patients ranked their satisfaction with the cosmetic outcome of their surgery below this threshold. There was no statistically significant difference in scar satisfaction or strength satisfaction between genders or age groups.

## Discussion

In keeping with previous reports of disproportionate rate of surgical intervention in males [37, 38], our cohort gender distribution is similarly skewed. Our findings demonstrate a higher rate of sensory disturbances following clavicle fracture fixation than previously reported in the literature. At an average of 19 months post-operatively, 68% of our cohort experienced ongoing paresthesia. Fifteen studies, including 836 patients, reported on average, when weighted, a 27% incidence of numbness at approximately 28 months following longitudinal approach to plate fixation of acute clavicle diaphyseal fractures. (Appendix I) [11, 19, 23–36]. However, this rate increases to 52% when the affected area is measured as part of the study protocol suggesting a possibility of under reporting [29, 34, 36]. We report a larger affected size (36.6 cm^2^ [+/− 26.9 cm^2^]) than the weighted average of 26 cm^2^ previously reported in the literature, though the variability seen in our findings represents the challenge inherent to patients’ self-estimation. Incision length was also not measured. Since our data represents a collection of surgeons, it may better represent real-world findings.

Over two thirds of our patients experienced some degree of improvement in both intensity and affected area which is consistent with previously reported trend of sensory improvement over time [23, 28, 29, 33, 35] though the underlying mechanism remains unclear (reinnervation by proximal injured branches or collateral branches). Shukla and co-workers [19] reported an association between perceived cosmetic outcome and overall satisfaction. This finding was replicated in our results though all three satisfaction sub-categories (range of motion, shoulder strength, and scar appearance) were significantly positively correlated with overall satisfaction emphasizing the concomitant importance of functional outcomes to patients. Similarly to previous work, we did not identify a relationship between cohort demographics (gender & age) and objective functional score [31], incidence of numbness, or cosmetic satisfaction [32]. Wang and co-workers [33] suggested that women could be more bothered by numbness due to sensory alteration affecting the breast and strap type clothing. While we identified a trend towards decreased cosmetic satisfaction in our female patients, we did not find a difference in overall satisfaction between genders as previously reported by Huang and colleagues [35]. Also contrary to Huang’s report, we also found increased numbness in women though this did not reach significance in our cohort likely due to the small number of women included in our study.

Our study is the only published report to assess the nature of sensory disturbances and 8% of our cohort reported tingling, burning, or pain without numbness. It is also the only study that demonstrated statistically significant worse subjective outcomes in patients who experienced a burning sensation. This may be related to neuroma formation [39]. Given the impact of sensory disturbance on patient outcomes, recent studies have explored alternative incision placements as well as minimally invasive plate osteosynthesis (MIPO) approach. Vertical incision, oblique incision following the nerve course longitudinally, or “necklace” incision following Langer’s lines have not consistently shown improved outcomes [26, 27, 33, 35]. MIPO technique, which utilizes the peripheral “safe zones” of the supraclavicular nerve path, has been shown to decrease the area size affected by paresthesia [29, 30, 36] without improvement in functional scores, operative time, union rate compared to traditional techniques, though there is some evidence of reduction in post-operative pain and improvement in patient satisfaction. This approach would lead to a smaller scar thus supporting our findings that cosmesis is related to patient satisfaction. Our results indicate that neuropathic pain significantly impacts patient satisfaction. A MIPO approach may reduce the risk of traumatic sharp injury to the nerve leading to neuroma formation. Further studies assessing the impact of MIPO technique on the development of neuropathic pain may better determine its association with improved outcomes to suggest change in traditional open approach techniques overlying the fracture entirely.

## Limitations

This retrospective observational study was not designed with an intention to assess a specific association but rather to evaluate overall general patient experience following clavicle fracture plate fixation. Therefore, the cohort size was arbitrarily defined and there was no control group which may introduce selection bias. Due to the small patient group, it is possible that some trends identified in our analyses represent true correlations, but significance could not be established. A minimum follow-up period of 1 year was used to ensure stabilization of neural injury and maximize the potential for recovery though later sensory changes could occur [31]. The eligibility criteria attempted to remove confounding surgical factors by excluding revision/repeat surgery and fixation following non-union which could impact supraclavicular nerve function and patient satisfaction. However, satisfaction and subjective function scores may be skewed by removing patients who experienced hardware irritation or continue to experience discomfort following removal due to neuroma formation, painful fracture site, or other.

Survey design was selected as the intent of the study was to establish subjective patient experience. In order to maximize the response rate, patients were not required to present themselves for an objective assessment (e.g. such as a grid measurement of affected area or two-point discrimination testing) nor were they required to complete objective functional scoring. This method inherently introduces response bias as patients were asked to recall events > 14 months prior to survey completion and patients who are dissatisfied with the result may have been less receptive to the request to complete the questionnaire. The survey did not assess for degree of fracture displacement/comminution, open/close nature of injury, pre-operative neurovascular status, mechanism of injury, patient handedness, patient baseline function/employment both of which could impact satisfaction measurements through confounding factors such as injury force, ipsilateral limb injury, ability to participate in rehabilitation and strength expectations/patient definition of function. While all fractures included in the study reached radiographic union, reduction/shortening was not assessed and could be a confounder in shoulder strength/overall satisfaction [37].

## Conclusions

Incisional paresthesia is very common after plate fixation of clavicle fractures. Most patients experience some improvement after a minimum of 1 year, though the majority experience persistent symptoms. There is significant variability in the area affected and degree of recovery. Numbness is the most common neurologic symptom but burning, when it occurs, is associated with lower patient satisfaction scores. Though rare, patients who experienced worsening intensity of symptoms were also less satisfied. Regardless of symptom severity or type, patients who were informed of the nature of this common complication fared better post-operatively.

## Supplementary Information

**Additional file 1**: **Appendix I**: Reported Incidence of Sensory Changes in the Supraclavicular Nerve Distribution following Longitudinal Approach to the Clavicle. **Appendix II**: Inclusion & Exclusion Criteria. **Appendix III**: Questionnaire.

## Data Availability

The datasets generated and/or analysed during the current study are not publicly available due to patient personal health confidentiality agreement but are available from the corresponding author on reasonable request.
