# Supplementary material for: Incisional paresthesia following clavicle plate fixation: does it matter to patients?
Source: BMC Musculoskelet Disord. 2021 Nov 3;22:928. doi: 10.1186/s12891-021-04770-z (PMC8567600; doi:10.1186/s12891-021-04770-z)
Supplement: Supplementary file 1 — Additional file 1: Appendix I: Reported Incidence of Sensory Changes in the Supraclavicular Nerve Distribution following Longitudinal Approach to the Clavicle. Appendix II: Inclusion & Exclusion Criteria. Appendix III: Questionnaire. [file 12891_2021_4770_MOESM1_ESM.docx]

SUPPLEMENTAL INFORMAITON:

**APPENDIX I: Reported Incidence of Sensory Changes in the Supraclavicular Nerve Distribution following Longitudinal Approach to the Clavicle**

*Note: The comprehensiveness of the literature review of all outcomes may be limited since sensory changes are often reported in the bulk of the results rather than as a main outcome listed in the searchable fields. Several studies described various time points or types of procedures, as we are most interested in final resolution of common plating with longitudinal incision, we have tabulated only the longest-reported follow-up of patients who underwent fixation mode of interest.*

| Authors | | Incidence (%) | Affected Area  (cm^2^) | Number of Patients | Mean Follow-up  (months) |
| --- | --- | --- | --- | --- | --- |
| Ou, et al., 2018 | | 1.5 | -- | 135 | 21.6 |
| Ferran, et al., 2010 | | 7 | -- | 15 | 12.4 |
| Shen, et al., 1999 | | 12 | -- | 232 | 52.8 |
| Ankers, et al., 2021 | | 23.5 | -- | 51 | 5.6 |
| COTS, 2007 | | 29 | -- | 62 | 12 |
| Li, et al., 2019* | | 12.5 | -- | 64 | 32.4 |
| Wang, et al, 2014 | | 36.8 | -- | 38 | 23.2 |
| You, et al., 2018 | | 37.1 | 17.89+/-12.09 | 35 | 12 |
| Kundangar, et al., 2019 | | 43 | -- | 16 | 24 |
| Chistensen, et al., 2014 | | 52 | 15 | 23 | 12 |
| d'Heurle, et al., 2013 | | 54.1 | -- | 37 | 31.6 |
| Wang, et al., 2010 | | 62 | -- | 21 | 12-36** |
| Chechik, et al., 2019 | | 69 | 48 ± 28 | 16 | 9.2 |
| Huang, et al., 2021 | | 70 | -- | 99 | 16 |
| Berier, et al., 2015 | | 74 | 19.8 ± 17.0 | 12 | 6 |
| Shukla, et al., 2017 | | 90*** | -- | 20 | 24.4 |
|  | **Area subgroup** | 52 | 26 | 63 | 10 |

*included middle and lateral third fractures

**range quotes, mean not provided - median of 24 months used for average calculation

*** represents all post-operative numbness and therefore study excluded from final weighted average; there is no distinction between early and late numbness incidence; severity is 1.9+/-0.7 at follow-up time

References: 9, 17, 21- 34

**APPENDIX II: Inclusion & Exclusion Criteria**

Inclusion Criteria:

- Clavicle Open Reduction and Internal Fixation (ORIF) with plate for midshaft or lateral clavicle fractures
- Timing of surgery less than 6 weeks post injury
- Age >18 years
- Ability to provide verbal consent
- Operation >1 year from day of study enrolment

Exclusion Criteria:

- Clavicle ORIF with plate for medial clavicle fractures
- Delayed ORIF for failed conservative management of fracture
- Revision clavicle ORIF
- Previous surgical intervention in the ipsilateral clavicle, shoulder, or upper anteromedial chest

**APPENDIX III: Questionnaire**

1. After the first surgery of your clavicle, have you had any other surgery on the same shoulder?
   - Yes/ No
2. Before the surgery, were you warned about the possibility of numbness (reduced or abnormal tingling/burning sensation) of the shoulder area?
   - Yes/No
3. Did you ever have any decreased or abnormal sensation around your incision after your clavicle surgery?
   - Yes/No
4. Do you currently have those symptoms? If not, when did you stop noticing these symptoms?
   - Ongoing vs. (numerical value) months after surgery
5. Please characterize the degree of loss of sensation around your shoulder from 0-10, where 0 indicates no loss of sensation and 10 indicates severe abnormal sensation.
   - 1-10, 0 = no loss of sensation, 10= Severe abnormal sensation
6. What is the character of your decreased or abnormal sensation?
   - Numbness, Tingling, Burning, Painful
7. Can you imagine how big a Canadian Quarter is? If not, do you have one near you? Can you tell me how many quarters would fit in the area of numbness/tingling/burning sensation right now?
   - Numerical value
8. Over time in the last year or so, has the size of the numbness/tingling/burning sensation decreased, stay the same, or increase?
   - Decreased, Unchanged, Increased
9. Over time in the last year or so, has the intensity of the numbness/tingling/burning sensation decrease, stayed the same, or increase?
   - Decreased, Unchanged, Increased
10. On a scale of 0-10 (where 10 is completely satisfied), how satisfied are you with the overall outcome after clavicle surgery?
    - 0-10, 0 = not satisfied, 10 = very satisfied
11. On a scale of 0-10 (where 10 is completely satisfied), how satisfied are you with the range of motion of your shoulder on the same side as your clavicle injury?
    - 0-10, 0 = not satisfied, 10 = very satisfied
12. On a scale of 0-10 (where 10 is completely satisfied), how satisfied are you with the strength after your clavicle surgery?
    - 0-10, 0 = not satisfied, 10 = very satisfied
13. On a scale of 0-10 (where 10 is completely satisfied), how satisfied are you with the scar after your clavicle surgery?
    - 0-10, 0 = not satisfied, 10 = very satisfied
